# Exploring the trajectory and correlates of social isolation for veterans across a 6-month period during COVID-19

**DOI:** 10.1371/journal.pone.0281575

**Published:** 2023-03-01

**Authors:** Adam P. McGuire, Colby Elmore, Yvette Z. Szabo, A. Solomon Kurz, Corina Mendoza, Emre Umucu, Suzannah K. Creech

**Affiliations:** 1 VISN 17 Center of Excellence for Research on Returning War Veterans, Waco, TX, United States of America; 2 Central Texas Veterans Health Care System, Temple, TX, United States of America; 3 Department of Psychology and Counseling, The University of Texas at Tyler, Tyler, TX, United States of America; 4 Department of Health, Human Performance and Recreation, Baylor University, Waco, TX, United States of America; 5 Department of Counseling, Educational Psychology, and Special Education, Michigan State University, East Lansing, MI, United States of America; 6 Department of Psychiatry and Behavioral Sciences, Dell Medical School of the University of Texas, Austin, TX, United States of America; La Trobe University - Melbourne Campus: La Trobe University, AUSTRALIA

## Abstract

Social isolation is a relevant problem for veterans who are at risk for disengaging from others as a function of transition stress from military life to civilian life, and given high rates of exposure to trauma and psychological distress. Few researchers have examined social isolation in veterans over time, particularly during COVID-19 that led to significant barriers and restrictions on social interactions. The purpose of this longitudinal study was to assess veterans’ experience of social isolation and its mental health and social functioning correlates during a 6-month period of the COVID-19 pandemic. Participants were 188 United States veterans of the Iraq and Afghanistan wars. A total of four assessments were administered: one every two months for a total duration of six months. The average number of completed assessments across all participants was 3.70 (*SD* = 0.75) with 159 participants (84.13%) completing all four timepoints. Surveys included measures of global mental health and social functioning as indicated by perceived emotional support, quality of marriage, and couple satisfaction. Multilevel modeling was used to assess 1) growth models to determine whether social isolation changed over time and the trajectory of that change (i.e., linear or quadratic); and 2) whether social isolation was related to both concurrent and prospective indicators of mental health and social functioning. All analyses included person mean centered and grand mean centered isolation to assess for within-and between-person effects. Veterans reported a quadratic trajectory in social isolation that decreased slightly and stabilized over time. Findings indicate that higher social isolation, at both the within- and between-person level, was negatively associated with concurrent emotional support, mental health, quality of marriage, and couple satisfaction. However, all prospective effects were nonsignificant at the within-person level. Results suggest although isolation may decrease over time, veterans report worse mental health and social functioning during times when they report higher levels of social isolation compared to themselves and others. Future work is needed to determine if interventions can be applied during those times to prevent or target those negative associations.

## Introduction

### Social isolation and veterans

Social isolation refers to the voluntary or involuntary absence of contact with others, which is associated with various negative consequences [1] and is a relevant problem for veterans in particular. Previous research indicates veterans are at risk for experiencing elevated levels of social isolation upon ending their military service and after re-entry into civilian life [2]. For example, one population-based study of older veterans found 44% reported feeling lonely at least some of the time and 10% reported feeling lonely often [3]. Accordingly, there are notable efforts to reintegrate veterans into civilian life and into their communities because of these observed risks for isolation [4]. Veterans are also at risk for mental health disorders due to high prevalence of exposure to trauma [5–7]. Thus, veterans may experience increased vulnerability to the negative effects of social isolation, which can be directly linked to mental health symptoms [8]. For example, social isolation can be used as a coping strategy to avoid perceived threatening scenarios, interpersonal conflict or discomfort, or anxiety in general [9].

To compound the issue, the presence of the coronavirus (COVID-19) led to a context in which the entire population, including veterans, were encouraged to physically isolate themselves from others for their own safety. Despite the opportunities for social connection that are afforded by technology, there is evidence that the safety concerns and physical limitations of COVID-19 were associated with higher degrees of perceived social isolation compared to typical levels outside of the pandemic [10]. Recent research on veterans further supports this claim as some studies indicate veterans are at increased risk of perceived social isolation due to the stressors of family and social relationship difficulties, boredom, and health difficulties [11]. Furthermore, some studies have noted that veterans are at increased risk for acute stress disorder associated with COVID-19 [12], which may lead to further isolation and avoidance. Altogether, these factors indicate veterans may be at heightened risk for social isolation and its negative effects since the start of the pandemic.

### Correlates of social isolation

In addition to the prevalence and risk for isolation, it is important to understand how veterans might experience isolation because of the known correlates to mental health and social functioning. Past research suggests social isolation is associated with a range of mental health issues including depression, anxiety, and suicidal ideation [13,14], issues with family and social relationships [11], and is associated with higher health care utilization [1]. Furthermore, people with preexisting mental health conditions are at increased risk for feelings of loneliness during social isolation. One large panel study found that people with psychiatric diagnoses were five times more likely to be categorized as *highly lonely* compared to those without a diagnosis [15]. Conversely, past studies have also found the presence of social connection can be a protective factor in reducing depressive and anxiety symptoms during the pandemic [16]. Living in rural areas, living with others, having a large group of close friends, and perceived social support have also been identified as protective factors against feelings of loneliness [15].

Regarding social functioning, specific facets of social support may also be important to consider, such as emotional support, which includes some of the intangible, relational features of support that could be conceivably impacted by social isolation [17]. Although social support overall is widely considered a protective factor for veterans [18,19], it is unclear how isolation in the context of COVID-19 is related to perceived emotional support. Additionally, it is important to understand how social isolation relates to perceived relationship quality and satisfaction among veterans who are involved in an intimate relationship; particularly during COVID-19 with a dearth of evidence on how isolation impacts relationships within this population. One study indicated that relationship quality and satisfaction can be tied to mental health during isolation [20], further emphasizing the potential impact of understanding this link during COVID-19. Given the potential correlates of poorer mental health and impaired social functioning, it is important to better understand the experience of social isolation in veterans.

### Longitudinal assessment of social isolation

Another important consideration for social isolation is the extent to which it changes over time, particularly for veterans during the course of COVID-19, and how within-person variability in isolation impacts the known correlates of mental health and social functioning. Given that social isolation is not an innately stable or static characteristic, and one’s level of isolation could vary over time depending on a wide range of circumstances, it follows that experiences of isolation could change. Presumably, the changes in social isolation could also have variable impact on mental health and social functioning at different times in a veteran’s life. However, few studies have assessed repeated measures of social isolation specifically despite the potential to expand our understanding of social isolation trajectories, which may inform possible resource allocation, support, and intervention for those struggling with isolation. Several studies have used longitudinal designs to examine loneliness in civilian and military populations with mixed findings that suggest variable trajectories over time [15,21–23]; however, loneliness is a distinct construct from social isolation that refers to the subjective distress from deficiencies in social relationships, rather than the objective absence of social contact that defines social isolation [8]. Furthermore, few studies have used repeated measures designs to disentangle the between- and within-person effects of social isolation on relevant outcomes. Given the potential for variability, it is important to understand how within-person differences in social isolation for veterans is related to mental health and social functioning. This investigation would expand on previous research that has primarily examined between-person differences in social isolation and known correlates.

### Current study

The purpose of this longitudinal study is to examine veterans’ experience of social isolation and its mental and social functioning correlates during a 6-month period in the early stage of the COVID-19 pandemic. First, we aimed to assess the trajectory of experienced social isolation using four repeated measures over six months. Second, using multilevel modeling, we aimed to examine how between-person and within-person differences in social isolation relate to global mental health and social functioning as indicated by perceived emotional support and relational factors (romantic relationship quality and couple satisfaction). Specifically, we examined both concurrent and prospective associations of social isolation on mental health and social functioning variables.

## Method

### Participants

One hundred and eighty-eight U.S. veterans of the wars in Iraq and Afghanistan were recruited from an ongoing longitudinal cohort study to participate in this 6-month longitudinal assessment study aimed at understanding the impact of the COVID-19 crisis on the mental health and functioning of veterans with and without pre-existing mental health difficulties. See Table 1 for the demographic characteristics of this sample.

**Table 1:** Demographic characteristics.

		*M* (*SD*) or *n* (%)
Age		46.93 (9.18)
Gender		
	Male	133 (70.37%)
	Female	55 (29.10%)
	Transgender (Female to Male)	1 (0.53%)
Sexual Orientation		
	Heterosexual	178 (94.18%)
	Bisexual	6 (3.17%)
	Homosexual	2 (1.06%)
	Questioning	1 (0.53%)
	Chose not to answer	2 (1.06%)
Race		
	White	112 (59.26%)
	Black or African American	68 (35.98%)
	American Indian or Alaska Native	17 (8.99%)
	Native Hawaiian or Pacific Islander	6 (3.17%)
	Asian or Asian American	2 (1.06%)
	Other	5 (2.65%)
Hispanic		27 (14.29%)
Education years		14.89 (2.33)
Education degree		
	High school/GED	9 (4.76%)
	Technical school certification	6 (3.17%)
	Some college, no degree	52 (27.51%)
	Associate’s degree	39 (20.63%)
	Bachelor’s degree	42 (22.22%)
	Some graduate school	12 (6.35%)
	Graduate degree	29 (15.34%)
Relationship status		
	Married	131 (69.31%)
	Single, in a relationship	26 (13.76%)
	Single, no relationship	19 (10.05%)
	Divorced	11 (5.82%)
	Widowed	1 (0.53%)
Income		
	$0 - $14,999	15 (7.94%)
	$15,000 - $29,999	21 (11.11%)
	$30,000 - $44,999	41 (21.69%)
	$45,000 - $59,999	41 (21.69%)
	$60,000 - $74,999	29 (15.34%)
	$75,000 - $89,999	17 (8.99%)
	$90,000 or higher	22 (11.64%)

### Procedures

Study procedures were approved by the Central Texas Veterans Health Care System Institutional Review Board (IRB; Protocol Number: 00720) prior to contacting any participants. All study procedures were conducted remotely via an online survey platform. Participants provided consent virtually by first reviewing a standard consent form that was presented in the survey platform. Next, they completed a multiple choice question by selecting “*I have read and understand the study information provided*, *and I consent/agree to participate*.” or “*I do NOT consent/agree to participate*.” Those who selected the *do not consent* option were immediately removed from the survey. Those who provided consent were immediately presented with the first battery of self-report measures. Documentation of informed consent was waived by the overseeing IRB. Recruitment for this study occurred during an 8-week period in June/July 2020 when Coronavirus rates surged in Texas [24], peaking in the middle of our recruitment window with nearly 11,000 new cases in a single day. Participants were recontacted for additional assessments every two months for six months for a total of four assessment timepoints.

### Measures

#### Social isolation

Social isolation during COVID-19 was measured using the National Institute of Health Patient-Reported Outcomes Measurement Information Systems (NIH PROMIS^®^) Social Isolation–Short Form [25]. Participants responded to a 4-item self-report measure of social isolation in which they were asked to select the frequency with which they experienced feelings of social isolation on a 5-point Likert scale (1 = *Never*, 5 = *Always*). Items were summed to create a total score, with higher scores indicating greater feelings of social isolation. Reliability estimates are included in Table 2.

**Table 2:** Descriptive and inferential statistics.

			Variance			
Variable	*Mean* (*SD*)	Range	Between	Within	Proportion Within	Reliability
Social Isolation	10.59 (4.72)	4–20	0.36	0.27	.43	.92
Emotional Support	14.90 (4.29)	4–20	0.21	0.30	.59	.90
Global Mental Health	5.39 (1.98)	2–10	0.36	0.09	.20	.91
Relationship Quality	34.78 (8.45)	6–45	1.41	0.75	.35	.81
Couple Satisfaction	29.68 (9.13)	0–41	0.43	0.23	.34	.93

#### Emotional support

Emotional support was measured using the NIH PROMIS^®^ Emotional Support–Short Form [25]. Participants responded to a 4-item self-report measure in which they were asked to select the frequency of feeling emotionally supported on a 5-point Likert scale (1 = *Never*; 5 = *Always*). Items were summed to create a total score, with higher scores indicating greater feelings of emotional support.

#### Mental health

Participants rated their mental health using the two-item NIH PROMIS^®^ Global Mental Health–Short From [25], which assesses mental health, mood, ability to think, and satisfaction with social relationships on a 5-point Likert scale (1 = *Never*; 5 = *Always*). Items were summed to create a total score, with higher scores indicating better mental health.

#### Romantic relationship quality

Veterans were screened for relationship status by asking “*Would you say you are currently in a romantic relationship of any kind*?” at each time point. Veterans who responded *yes* to the screener question completed the 6-item self-report Quality of Marriage Index (QMI) used to measure the quality of a romantic relationship [26]. Each of the items was rated on 7-point Likert Scale (1 = *Very Strongly Disagree*, 7 = *Very Strongly Agree*). Items were summed to create a total score, with higher scores indicating better relationship quality.

#### Couple satisfaction

Participants who endorsed being in a current romantic relationship (see screener question above) completed the Couple Satisfaction Index-Short Form (CSI-SF), an 8-item self-report of relationship distress [27]. The anchors and framing of questions vary across items. Overall, items ask the participant to report on their happiness and agreement with a range of statements about relationships. Items are summed to create a total score ranging from 0 to 41, with higher scores indicating higher levels of relationship satisfaction.

### Data analytic plan

All data management and analyses were conducted with R [28]. First, we used the nlme package [29] to estimate reliability for all measures using unconditional linear mixed effects models. This method is recommended for nested data that violate the assumption of independence [30,31], which describes the current study’s use of repeated measurements across four timepoints. We used a three-level model structure with individual items for each measure (Level 1) nested within each measure’s total score (Level 2) nested within each person (Level 3). Reliability estimates were calculated using the variance at item, total, score, and person levels, which resulted in an estimate similar to Cronbach’s α that accounts for differences between repeated measurements and persons (Table 2).

Next, we examined growth models for social isolation using multilevel modeling with the nlme package. Prior to analysis, we calculated time for each of the four assessments to represent the specific number of days since the baseline appointment, which was coded as *time* = 0. All assessments were scheduled to be completed approximately 60 days apart from one another, but not all veterans responded on that exact day at each timepoint. We fit a linear growth model by adding raw time as a predictor of social isolation, which indicates whether social isolation increases or decreases over time. We fit a quadratic growth model by adding raw time and time^2^ as two predictors of social isolation. Raw time as a predictor still represents the extent to which social isolation changes over time (linear effect) and time^2^ represents the rate of change over time (quadratic effect). The anova function was used to compare the linear and quadratic growth models, with a significant likelihood ratio test and a lower Akaike information criterion (AIC) indicating which model fit the data better, while accounting for model complexity.

For concurrent and prospective models, we first calculated the average social isolation score for each person across all four timepoints (i.e., person mean), which represents between-person differences in social isolation among the sample. We also calculated person-centered isolation scores by subtracting the score at each timepoint from the person mean (i.e., person-centered), which represents within-person differences. We fit four concurrent models with emotional support, global mental health, romantic relationship quality, and couple satisfaction as the dependent variables. Each model included person-centered isolation (within-person effects), person mean isolation (between-person effects), and time (covariate) as predictors with random intercepts and slopes. We also estimated separate, prospective models with the same predictors, but lagged the score for each dependent variable such that social isolation at any given timepoint was predicting the dependent variable at the subsequent timepoint (e.g., Time 1 Isolation ◊ Time 2 Emotional Support). For the models that examined romantic relationship quality and couple satisfaction, we restricted the sample to participants who endorsed being in a current relationship at all four timepoints (*n* = 88; 46.81% of sample). This allowed for an assessment of between- and within-person effects across the entire 6-month period, and removed some of the potential confounds associated with starting or ending relationships during that time. All plots and figures were created with the ggplot function in the tidyverse package [32].

## Results

After completing the baseline assessment, the average number of days passed until subsequent timepoints include 68.01 days (*SD* = 12.93) for T2, 131.12 days (*SD* = 33.69) for T3, and 189.48 days (*SD* = 11.95) for T4. The average number of completed assessments across all participants was 3.70 (*SD* = 0.75) out of 4. A total of 159 participants (84.13%) completed all 4 timepoints. Unconditional models indicate that reliability estimates for all measures were acceptable (Table 2). Additionally, models suggest a higher proportion of variance for emotional support is attributed to within-person differences (59%), whereas between-person differences represent a higher proportion for social support, global mental health, romantic relationship quality, and couple satisfaction (57–80%). Means and standard deviations for all measures are also reported in Table 2.

### Growth models

**Fig 1:**
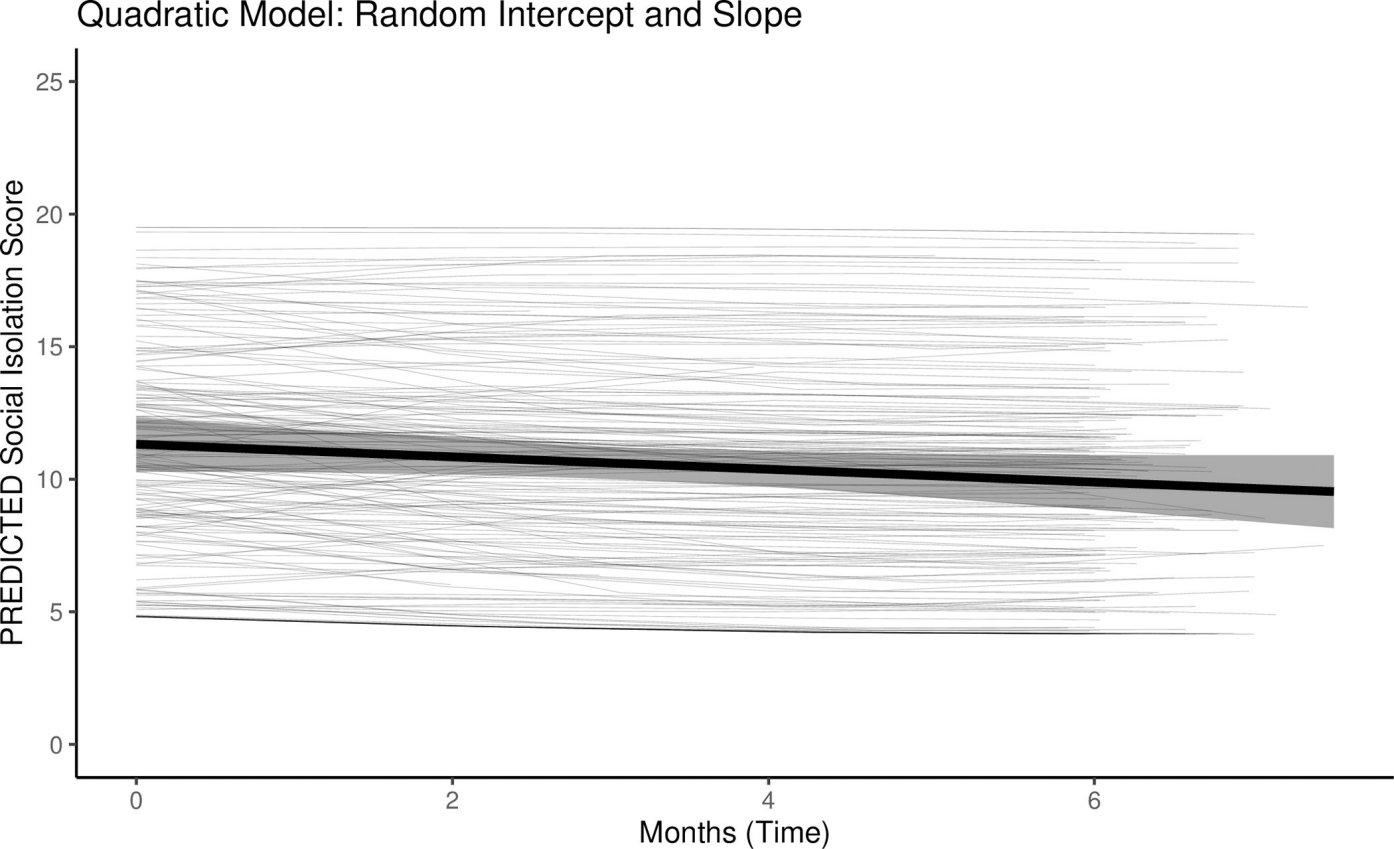


We modeled the growth trajectories of social isolation to see how much it tended to change within and between veterans over the 6-month assessment period. We also examined whether the change was best described as linear or nonlinear. In the linear growth model with raw time (number of days) as the only predictor, there was a significant, small negative association for time and social isolation (*b* = -0.72 [95% CI: -1.21, -0.24], *SE* = 0.25, *p* = .004). This result suggests a slight, linear decrease in isolation over the 6-month period. In the quadratic growth model with raw time and time^2^ as the two predictors, there was a negative, but nonsignificant effect for time represented as linear (*b* = -1.43 [-3.06, 0.21], *SE* = 0.83, *p* = .087) and a nonsignificant effect for time represented as quadratic (*b* = 0.67 [-0.74, 2.08], *SE* = 0.72, *p* = .350). Model comparison indicated the AIC was lower for the quadratic growth model (3653.32) compared to the linear growth model (3655.54), and the likelihood ratio test was statistically significant (*p* = .037) in favor of the quadradic model. Thus, the best fitting model indicated many had an initial decrease in social isolation that lessens in the rate of change over time (see Fig 1). However, the level-2 variance parameters indicated the veterans differed considerable in their trajectories.

### Concurrent and prospective effects

Last, we assessed for concurrent and prospective effects of social isolation across four different outcome variables: emotional support, mental health, romantic relationship quality, and couple satisfaction. For all models, person mean scores were entered as a predictor of between-person differences in isolation. Person-centered scores were entered as a predictor of within-person differences in isolation. Thus, coefficients for person-centered isolation represented changes in the outcome variable that are associated with deviations from a participant’s average level of isolation.

For concurrent models, both person-centered and person mean isolation were statistically significant predictors with negative coefficients across all models (see Table 3). Regarding person-centered effects, as participants reported higher levels of social isolation than their typical average level of isolation, they tended to report lower levels of emotional support, global mental health, couple satisfaction, and romantic relationship quality at that same timepoint. Notably, there was a stronger association with all outcome variables for person mean scores compared to person-centered scores.

**Table 3:** Results from concurrent and prospective analyses.

		Concurrent			Prospective		
Variable		Estimate [95% CI]	*SE*	*p*	Estimate [95% CI]	*SE*	*p*
*DV*: *Emotional support (n = 188)*							
	Person-centered	**-0.14 [-0.24, -0.03]**	**0.05**	**.009**	0.01 [-0.11, 0.13]	0.06	.898
	Person mean	**-0.54 [-0.64, -0.43]**	**0.05**	**< .001**	**-0.55 [-0.66, -0.44]**	**0.06**	**< .001**
*DV*: Global mental health* *(n = 188)*							
	Person-centered	**-0.14 [-0.18, -0.10]**	**0.02**	**< .001**	0.02 [-0.03, 0.06]	0.02	.484
	Person mean	**-0.32 [-0.36, -0.28]**	**0.02**	**< .001**	**-0.32 [-0.37, -0.28]**	**0.02**	**< .001**
*DV*: *Relationship quality (n = 88)*							
	Person-centered	**-0.58 [-0.89, -0.27]**	**0.16**	**< .001**	0.05 [-0.34, 0.45]	0.20	.785
	Person mean	**-0.77 [-1.10, -0.45]**	**0.16**	**< .001**	**-0.83 [-1.17, -0.50]**	**0.17**	**< .001**
*DV*: *Couple satisfaction (n = 88)*							
	Person-centered	**-0.36 [-0.62, -0.10]**	**0.13**	**.006**	-0.19 [-0.47, 0.09]	0.14	.185
	Person mean	**-1.03 [-1.40, -0.65]**	**0.19**	**< .001**	**-1.05 [-1.43, -0.66]**	**0.19**	**< .001**

*Note*. *Results represent a model with random intercept and fixed slope because the model with random slope failed to converge; Boldface indicates *p*-value < .05; CI = confidence interval; SE = standard error; DV = dependent variable.

For prospective models that examined the effects on lagged outcome variables, person-mean isolation was a significant predictor with a negative coefficient for all outcomes, consistent with the concurrent models. However, person-centered isolation at a given timepoint was small and not significantly associated with any outcome at the subsequent timepoint.

## Discussion

This longitudinal study aimed to examine veterans’ experiences of social isolation over six months during the COVID-19 pandemic. We assessed the trajectory of social isolation to determine the extent to which it changed over six months, and we evaluated how between- and within-person differences in social isolation were associated with concurrent and prospective mental health and social functioning. Overall, results demonstrate a small decrease in social isolation initially, but not continued decreases over time. In addition, higher social isolation in comparison to other participants was associated with lower global mental health, emotional support, relationship quality, and couple satisfaction. Importantly, results also indicated that deviations from one’s average level of social isolation over six months is associated with negative outcomes, suggesting avenues for potential prevention in the future.

### Social isolation trajectory

Our results demonstrated a small negative association for social isolation in the linear growth model, indicating that social isolation decreased over time at the group-level. However, the better-fitting quadratic growth model further suggests the decrease in isolation tended to stabilize over time as the rate of change lessens. Upon further inspection, there was noticeable variability in the quadratic term when slopes were set as random (i.e., free to vary from the aggregate slope). Although the population quadratic term (time^2^) was small and nonsignificant, the quadratic growth model demonstrated better fit because it allowed for differences among the veterans in how they changed over time. These results suggest future research should consider exploring individual factors that can help explain or predict differences in trajectories for this population.

The overall finding of slight decreases and stabilization of isolation is somewhat contradictory to concerns about community mitigation strategies (e.g., social distancing, lockdown) to reduce and/or prevent transmission of COVID-19, and contradictory to reasonable expectations that social isolation might increase in veterans [33]. However, previous studies have also reported similar findings with regard to loneliness. For example, one civilian-based study examined loneliness trajectory in response to COVID-19 and found stable levels of loneliness and increased support from others over a 3-month period [22]. Another study found similar results that levels of loneliness remained stable in a community-dwelling sample of older adults over a period of 10 weeks during the pandemic [34]. Furthermore, isolation may have stabilized as COVID-19 mitigation strategies became more normalized and lock-downs eased.

Although the factors influencing social isolation in veterans is complex, and we do not have a civilian comparison sample, one potential explanation for this finding could be related to veterans’ previous military-related experiences. Veterans could have demonstrated a resilience to worsening isolation given prior experiences coping with social isolation before the pandemic—a prevalent issue for this population in general [8]. For example, veterans may have successfully used online social networking strategies that were previously relied upon to remain in contact with members of their military community before the pandemic. Previous work also reported that higher online connections were associated with lower psychological distress regardless of the levels of available face-to-face connections during the beginning of the lockdown [35]. Another possible explanation could be that veterans were able to rely on existing forms of social connection or membership with communities that buffered against feelings of isolation, despite COVID-19 mitigation strategies. Although deployment-related factors may trigger mental health challenges, research also revealed that factors such as social support, perceived comradeship during deployment, and the perception that deployment had a positive effect on one’s life are negatively related to loneliness [36]. Thus, deployment-related experiences that are considered positive or strengths could play a protective role for veterans. Although we did not take these deployment-related factors into account, future studies should further investigate their potential influence on isolation over time.

Although social isolation seems relatively stable in this veteran sample, it is unclear how this trajectory was impacted by the pandemic itself or other unknown factors. For example, this study took place during the beginning of the COVID-19 pandemic, but it would be helpful to understand how social isolation is experienced as the pandemic and mitigation strategies continue beyond the six months examined. Additionally, for this specific veteran population, it would be important to better understand how social isolation is experienced over a period of time associated with discharge from the military or attempts to reintegrate into civilian life. Therefore, future research should aim to use similar methods of repeated assessment to assess experiences and potential changes in social isolation within specific contexts relevant to the life experiences of veterans and servicemembers.

### Concurrent effects

Leveraging a repeated measures design, we examined whether within- and between-person differences in social isolation were associated with mental health and social functioning variables. Both within- and between-person effects of social isolation were negatively and significantly associated with all outcome variables in concurrent models.

First, higher isolation in comparison to other participants was linked with lower global mental health, emotional support, relationship quality, and couple satisfaction. This is consistent with previous research that found the related construct of loneliness was negatively correlated with social support and positively correlated with mental health symptoms, which demonstrated medium to large-sized effects [3,21]. Previous studies examining social disconnection also found similar results with poorer social functioning [17] and relationship quality [37]. However, results from this study expand on those findings by demonstrating similar effects across participants (i.e., between-person effects) for social isolation specifically in a sample of military veterans.

Additionally, a unique contribution of these findings is the within-person effects that examines social isolation as a dynamic variable that can fluctuate over time. Specifically, the models indicate that regardless of between-person differences (i.e., their average level of isolation over six months), as veterans reported more isolation than what was typical for themselves, they experienced lower emotional support, mental health, relationship quality, and couple satisfaction. Conversely, isolation at one measurement period that was lower than one’s typical level of social isolation was linked to higher mental health and social functioning. These findings are consistent with between-person effects as described; however, there is a dearth of research on the within-person effects of isolation. One exception is with loneliness and similar relationship variables. Results from the current study are consistent with another study that also examined within-person effects of loneliness in partners during COVID-19 and found person-centered loneliness predicted lower relationship satisfaction and more conflict in a civilian sample [38]. Therefore, this study offers an important contribution by expanding our understanding of the within-person effects of social isolation across these mental health and social correlates, particularly among veterans.

With additional support, these findings could be important to consider in efforts to assess and intervene on isolation or a lack of connectedness within this population. For example, for those who are interested in enhancing connection and community integration for veterans, this study supports the need for repeated assessments of social isolation given that deviations from one’s average level over six months is associated with negative outcomes. Capturing those moments when veterans are particularly more isolated than what is normal for them could lead to preventative efforts, whereas identifying moments of lower-than-normal isolation might indicate moments of personal strength or security that could be leveraged to facilitate person-centered goals. Additionally, these findings highlight the importance of exploring intervention efforts that might help veterans reduce levels of isolation beyond their typical experience (i.e., activating deviations below person-mean isolation). A recent systematic review found several therapy-based interventions focusing on mindfulness or meditation have shown promise in reducing loneliness in civilian populations, yet studies that attempted to reduce social isolation reported mixed findings [39]. Therefore, more research is needed to test the feasibility and effectiveness of different isolation-reducing strategies with veterans, such as enrolling in mentoring programs, participating in group-based volunteer work, or joining veteran or community-wide organizations that involve face-to-face interactions and facilitate greater social engagement.

### Prospective effects

Regarding prospective analyses, there were no significant within-person effects during this 6-month period, which suggests social isolation at one timepoint was not predictive of delayed experiences of outcome variables approximately two months later. Although person mean isolation was significant for prospective models, those effects represent the relation between *aggregate scores* of social isolation across all timepoints and each outcome, whereas person-centered effects represent the relation between a social isolation score for an individual timepoint and a score for an outcome variable at the subsequent timepoint. Thus, person mean predictors do not indicate true prospective effects and are primarily used to account for between-person variability in these prospective models. The distinction between significant concurrent effects and nonsignificant prospective effects demonstrates the utility of repeated measurements in understanding the long-term impact of social isolation. Specifically, it helps contextualize the concurrent effects so that we do not jump to causal interpretations that within-person deviations in isolation automatically translates to subsequent changes in future outcomes. However, the null findings also raise questions about the mechanisms linking the significant relationship between concurrent isolation and mental health and social functioning. Perhaps there are additional personal characteristics or situational factors that contribute to these relationships, which should be explored in future research. Alternatively, the absence of a prospective effect between two measurements roughly 60 days apart may suggest more work is needed to understand whether social isolation has a more immediate (e.g., the next day or week) versus long-term impact on outcomes. Future research should continue to use longitudinal designs with different assessment schedules to fully explore the potential impact of social isolation and to better understand how, if at all, it leads to negative outcomes in the immediate future.

### Limitations

Although this study has significant strengths, it is important to highlight several limitations. First, there is no data available for social isolation prior to COVID-19, which could impact both trajectory and isolation correlates. Second, assessments included brief measures that might not fully capture the experience of social isolation, emotional support, and global mental health. Brief measures were important in this longitudinal design to reduce participant burden, but future research would benefit from using more comprehensive measures to assess these constructs. Third, participants retrospectively rated all measures as they experienced items over the past month, which could be biased by poor recall or their current state. Fourth, this sample included veterans with and without clinical symptoms, but it’s unclear how the findings might vary in those with differing levels of underlying psychological distress. Furthermore, social isolation may play different roles in veterans with underlying symptoms that could moderate some associations. For example, veterans with severe PTSD symptoms may have perceived isolation as beneficial and may use it to alleviate distress in the short-term despite long-term consequences. Future work should aim to understand the unique impact of these processes on those with and without significant psychological distress. Although these findings provide useful insight on experiences of social isolation over time during the pandemic, the extent to which these results apply to veterans outside the context of this ongoing stressor is also unknown. Additional longitudinal studies are needed to monitor social isolation after the pandemic to better understand how veterans experience isolation during salient times for that population (e.g., return from deployment, life transitions, etc.). Finally, social isolation is a multidimensional construct that is related to several situational factors and overlapping constructs such as loneliness. Future research might need to assess different domains of social isolation and explore the nuanced differences between isolation and loneliness to better understand specific factors that are associated with negative or positive outcomes.

### Conclusions

This study suggests that veterans’ experience of social isolation during the initial stage of the COVID-19 pandemic decreased slightly and was relatively stable over time. Notably, results also indicate that deviations from average levels of isolation across the population and deviations from one’s typical level of isolation are associated with worse mental health and social functioning outcomes. Given the importance of increasing social engagement and community reintegration for veterans in particular, future work should expand on these findings to determine if enhanced assessment over time and potential intervention for social isolation could be implemented to prevent or target these negative associations.
